# Etiologic distribution and clinical characteristics of pediatric diabetes in 276 children and adolescents with diabetes at a single academic center

**DOI:** 10.1186/s12887-021-02575-6

**Published:** 2021-03-04

**Authors:** Ja Hye Kim, Yena Lee, Yunha Choi, Gu-Hwan Kim, Han-Wook Yoo, Jin-Ho Choi

**Affiliations:** 1 Department of Pediatrics, Medical Genetics Center, Asan Medical Center, University of Ulsan College of Medicine, 88, Olympic-Ro 43-Gil, Songpa-Gu, Seoul, 05505 Republic of Korea; 2 Medical Genetics Center, Asan Medical Center, University of Ulsan College of Medicine, Seoul, Republic of Korea

**Keywords:** Maturity-onset diabetes of the young, Monogenic diabetes, Type 1 diabetes mellitus, Type 2 diabetes mellitus

## Abstract

**Background:**

The prevalence of monogenic diabetes is estimated to be 1.1–6.3% of patients with diabetes mellitus (DM) in Europe. The overlapping clinical features of various forms of diabetes make differential diagnosis challenging. Therefore, this study investigated the etiologic distribution and clinical characteristics of pediatric diabetes, including monogenic diabetes, who presented at a single tertiary center over the last 20 years.

**Methods:**

This study included 276 consecutive patients with DM diagnosed before 18 years of age from January 2000 to December 2019 in Korea. Clinical features, biochemical findings, β-cell autoantibodies, and molecular characteristics were reviewed retrospectively.

**Results:**

Of the 276 patients, 206 patients (74.6%), 49 patients (17.8%), and 21 patients (7.6%) were diagnosed with type 1 DM, type 2 DM, and clinically suspected monogenic diabetes, respectively. Among 21 patients suspected to have monogenic diabetes, 8 patients had clinical maturity-onset diabetes of the young (MODY), and the remaining 13 patients had other types of monogenic diabetes. Among them, genetic etiologies were identified in 14 patients (5.1%) from 13 families, which included MODY 5, transient neonatal DM, developmental delay, epilepsy, and neonatal diabetes (DEND) syndrome, Wolfram syndrome, Donohue syndrome, immune dysregulation, polyendocrinopathy, enteropathy, X-linked (IPEX) syndrome, Fanconi-Bickel syndrome, Wolcott-Rallison syndrome, cystic fibrosis-related diabetes, and maternally inherited diabetes and deafness.

**Conclusions:**

Genetically confirmed monogenic diabetes accounted for 5.1% of patients evaluated at a single tertiary center over 20-year period. Based on the findings for our sample, the frequency of mutations in the major genes of MODY appears to be low among pediatric patients in Korea. It is critical to identify the genetic cause of DM to provide appropriate therapeutic options and genetic counseling.

**Supplementary Information:**

The online version contains supplementary material available at 10.1186/s12887-021-02575-6.

## Background

Among children and adolescents with diabetes mellitus (DM), type 1 DM is the most common, especially in North America and Europe [1]. However, the annual incidence of type 1 DM varies according to the ethnic background, and worldwide incidence has increased in the past two decades [1–3]. In Korea, the annual incidence of type 1 DM increased from 1.36/100,000 in 1995–2000 to 3.19/100,000 in 2012–2014 [4]. In addition, increasing worldwide rates of child obesity have been associated with a variable increase in the prevalence of type 2 DM depending on the ethnic background and region of residence [2].

Most DM cases are classified as type 1 or type 2 DM (i.e., of multifactorial etiology). Type 1 DM is primarily caused by the autoimmune destruction of pancreatic β-cells [5]. A substantial proportion of type 2 DM is caused by environmental and multiple genetic defects with a smaller effect size. Compared with type 2 DM, monogenic diabetes, including maturity-onset diabetes of the young (MODY), is a rare form of DM caused by mutations in one of more than 20 genes that control either the secretion or action of insulin [6]. Monogenic diabetes is characterized by Mendelian inheritance pattern with a large effect size of causal variants and minimal environmental contributions [7]. The prevalence of monogenic diabetes is estimated to be 1.1–6.3% among children and adolescents with DM in Europe [8–13].

It is important to make an accurate etiologic diagnosis of DM since it can affect the therapeutic decisions, the prognosis of chronic complications, and genetic counseling [14]. However, the overlapping clinical features of various forms of DM make it difficult to perform differential etiologic diagnosis. Information on the distribution of the type of DM and the prevalence of monogenic diabetes has not been established in the Korean pediatric population. A few studies on MODY in Korea demonstrated that mutations in the major MODY genes, including some cases with *GCK* mutations, were rare [15–18]. Therefore, this study was performed to investigate the etiologic distribution and clinical characteristics of pediatric diabetes including monogenic diabetes at a single tertiary center over a 20-year period in Korea.

## Methods/design

### Subjects

Three hundred and sixty-two patients diagnosed with DM before the age of 18 years who presented at the Department of Pediatrics, Asan Medical Center Children’s Hospital, or were referred from another hospital between January 2000 and December 2019, were included in the study. However, 47 patients with type 1 DM and 10 patients with type 2 DM who were referred from another hospital were excluded from the study owing to insufficient clinical and laboratory data, and 29 patients with secondary DM due to corticosteroid or immunosuppressant use were also excluded because these patients showed different clinical course of transient hyperglycemia. Ultimately, 276 consecutively presenting pediatric patients were included.

Clinical and endocrine characteristics such as the patients’ age at diagnosis, gender, C-peptide level, presence of β-cell autoantibodies (glutamic acid decarboxylase antibody and insulin autoantibody), and presence of diabetic ketoacidosis (DKA) at diagnosis, were retrospectively collected using a medical chart review. Laboratory tests were performed at the time of diagnosis at a single academic center.

Type 1 DM was diagnosed in the presence of low C-peptide levels, β-cell autoantibody positivity, and the absence of acanthosis nigricans or any extra-pancreatic features suggesting monogenic diabetes. The diagnosis of type 2 DM was based on clinical findings, such as a family history of type 2 DM, obesity, and signs of insulin resistance (acanthosis nigricans). The patients were categorized as having monogenic diabetes when they showed clinical features, such as a family history of DM, the absence of β-cell autoantibodies, normal or high C-peptide levels, a low-dose insulin requirement, and signs of extra-pancreatic features [14].

Four patients with monogenic diabetes previously reported by our group [19–22] were included to delineate the clinical and molecular spectrum of monogenic diabetes in the cohort included in the current research. This study was approved by the Institutional Review Board of Asan Medical Center, Seoul, Korea (IRB No. 2020–0667). Blood samples for DNA analysis were collected after obtaining written informed consent from the patients or their parents. Informed consent was obtained from both the parents and patient for participation by pediatric patients aged 7–17 years.

### Molecular analysis for patients with monogenic diabetes

Genomic DNA was extracted from peripheral blood leukocytes using the Gentra Puregene Blood kit (Qiagen, Hilden, Germany). Molecular analysis was performed by Sanger sequencing for patients with a clinical diagnosis of monogenic diabetes according to the patients’ phenotype. Sanger sequencing of the major genes (*HNF1A*, *HNF4A*, and *GCK* genes) involved in MODY was performed when MODY was clinically suspected: 1) a family history of diabetes in one parent and in a first-degree relatives of the affected parent; 2) negative autoantibodies; 3) lack of the characteristics of type 2 DM (obesity and acanthosis nigricans); and 4) good metabolic control with diet, sulfonylurea therapy, or low-dose insulin [14]. In cases with defects of the urogenital tract, *HNF1B* was analyzed. DNA sequencing of *INSR* was performed for a patient with severe insulin resistance.

For patients with MODY, whole exome sequencing was performed when there were no rare sequence variants in the major MODY genes. SureSelect Human All Exon V5 (Agilent Technologies, Santa Clara, CA, USA) was used for library preparation. Sequencing was performed using the NextSeq500 platform (Illumina Inc., San Diego, CA, USA), generating 2 × 150 bp paired-end reads. The sequence reads were aligned to the human reference genome (hg19) using the Burrow-Wheeler Alignment program (BWA version 0.7.12). SAMtools 0.1.19 and Genome Analysis Toolkits (GATK version 3.5) were used for single nucleotide polymorphism variant calling from aligned sequence reads. GATK version 3.5, FreeBayes 0.9.2.1, and Scalpel 0.5.3 were used for insertion-deletion variant calling. After removing duplicates with Picard (version 1.96), annotation was performed using Variant Effect Predictor [23] and dbNSFP [24]. The sequence variants of known genes previously associated with MODY were scanned [25]. All sequence variants were classified as pathogenic, likely pathogenic, variant of uncertain significance (VUS), likely benign, or benign, in accordance with the standards and guidelines of the American College of Medical Genetics and Genomics and the Association for Molecular Pathology [26].

## Results

### Clinical and molecular characteristics of patients with type 1 and type 2 diabetes mellitus

**Fig. 1:**
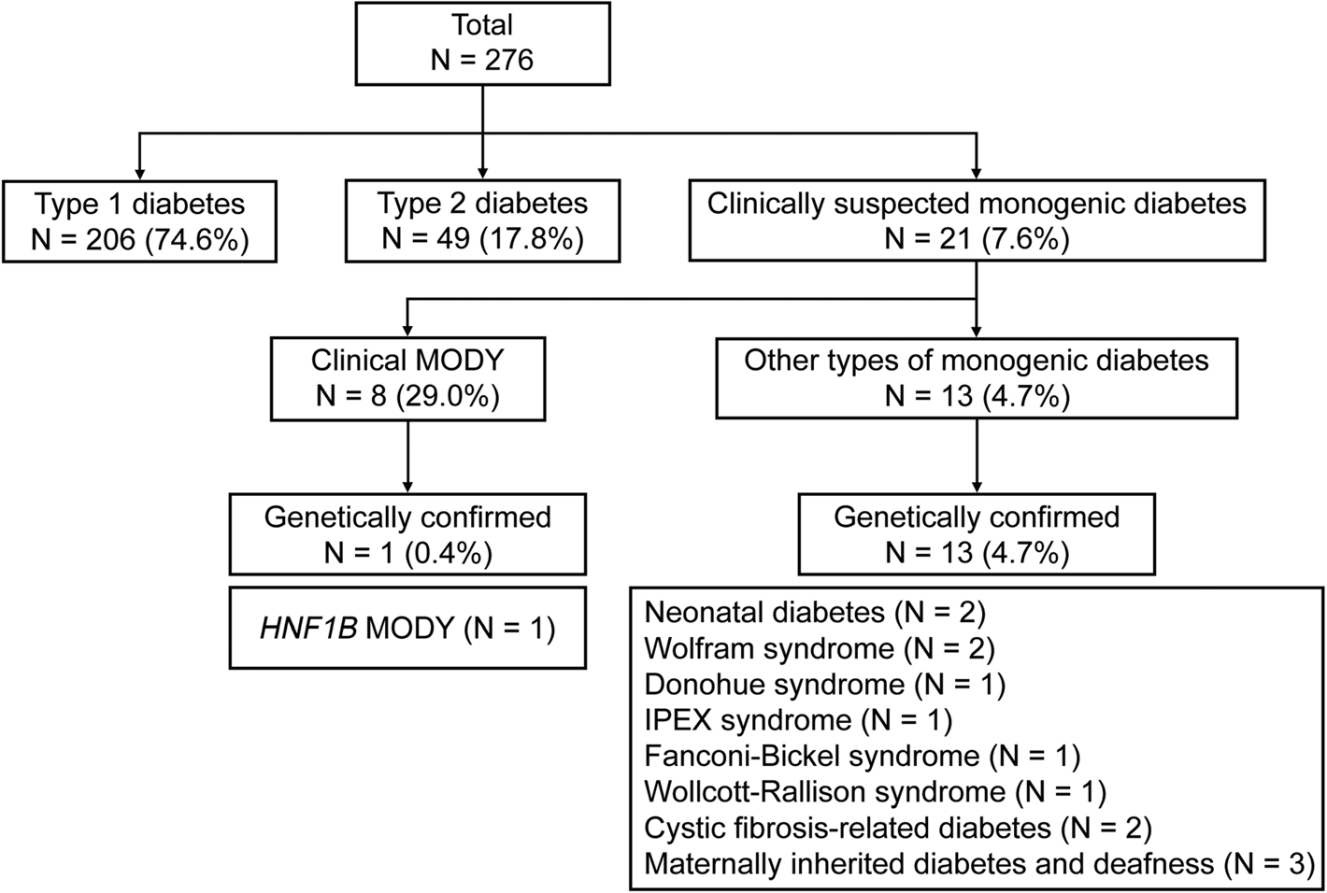
A flow chart illustrating the etiologic spectrum of pediatric diabetes mellitus (DM). MODY, maturity-onset diabetes of the young

**Fig. 2:**
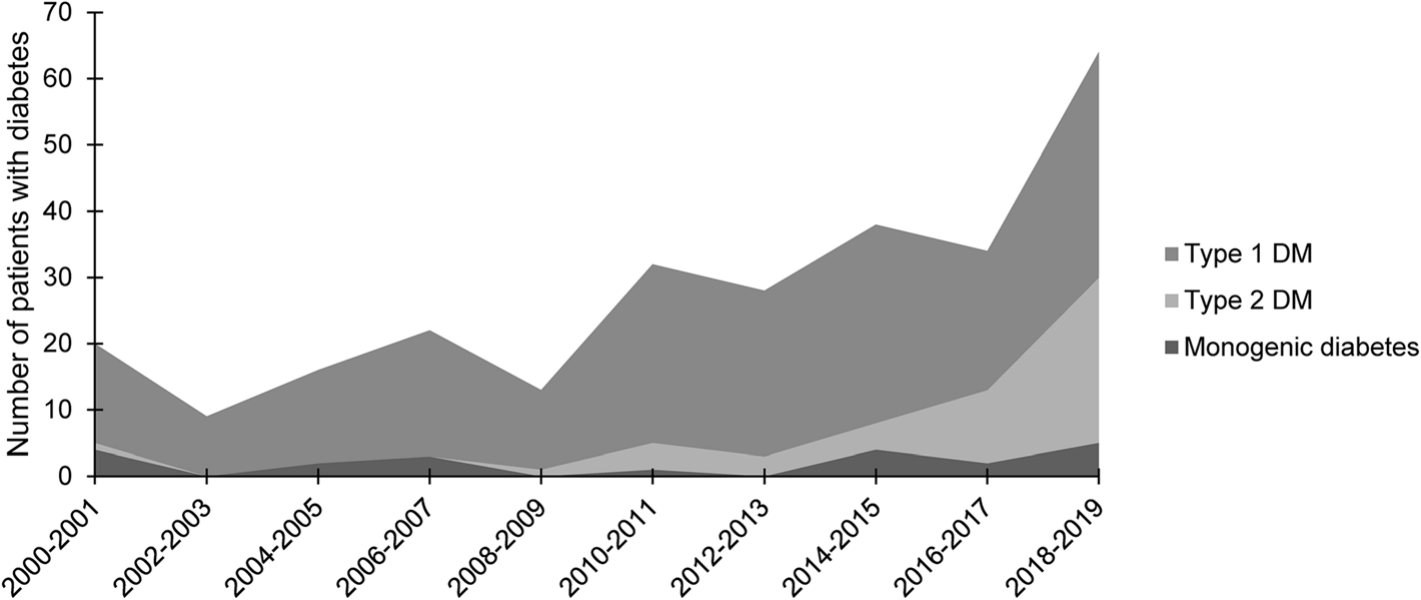
The annual frequency of diabetes mellitus (DM) by type in children and adolescents at a single academic center

Of the 276 patients, 206 (74.6%), 49 (17.8%), and 21 (7.6%) patients were diagnosed with type 1 DM, type 2 DM, and clinically suspected monogenic diabetes, respectively (Fig. 1). The clinical characteristics at diagnosis of type 1 DM, type 2 DM, and monogenic diabetes are shown in Table 1. Trend of frequency of new diabetes cases is detailed in Fig. 2; in particular, the frequency of type 2 DM increased during the study period.

**Table 1:** Clinical features of patients with diabetes mellitus (DM) at diagnosis

	Type 1 DM	Type 2 DM	MODY	Neonatal DM	CFRD	Genetic syndromes	MIDD
Patients, n (%)	206 (74.6)	49 (17.8)	8 (2.9)	2 (0.7)	2 (0.7)	6 (2.2)	3 (1.1)
Age at diagnosis, years	9.9 ± 3.8	13.7 ± 2.1	13.5 ± 2.8	0.1	10.6	2.1 ± 2.7	4.1 ± 0.9
BMI Z-score	−0.83 ± 1.22	2.59 ± 1.47	− 0.33 ± 1.00	NA	0.45 ± 4.01	NA	− 3.29 ± 2.05
HbA1c, %	12.1 ± 2.5	10.0 ± 2.0	9.7 ± 3.0	NA	6.9	9.4 ± 2.9	8.2 ± 2.8
C-peptide, ng/mL	0.9 ± 1.0	4.7 ± 2.7	2.2 ± 1.9	0.45	3.1	22.9 ± 38.8	6.3 ± 4.9
Antibody positivity, n (%)	137/190 (72.1)	7/49 (14.3)	0	0	0	0	0
DKA at diagnosis, n (%)	40 (19.4)	1 (2.0)	0	0	0	0	0

*BMI* body mass index, *CFRD* cystic fibrosis-related diabetes, *DKA* diabetic ketoacidosis, *HbA1c* hemoglobin A1c, *MIDD* maternally inherited diabetes and deafness, *NA* not available

Of the 206 patients with type 1 DM, the mean age at diagnosis was 9.9 ± 3.8 years (median, 10.4 years; range, 0.8–17.6 years). The frequency of DKA at diagnosis in type 1 DM participants was 19.4% (*n* = 40). The β-cell autoantibodies of 190 patients were analyzed. Among them, 137 patients (72.1%) had at least one β-cell autoantibody. The mean serum C-peptide level at diagnosis was 0.9 ± 1.0 ng/mL (reference range, 0.48–3.3 ng/mL).

Among 49 patients with type 2 DM (17.8%), the mean age at diagnosis was 13.7 ± 2.1 years (median, 13.5 years; range, 8.0–16.8 years). The mean fasting serum C-peptide level (available for 44 patients) was 4.7 ± 2.7 ng/mL. DKA and hyperosmolar hyperglycemic state were observed at diagnosis in 1 patient (2.0%) with type 2 DM. Antibody positivity was 14.3% (7 patients) in type 2 DM participants.

### Clinical and molecular characteristics of patients with genetically confirmed monogenic diabetes

Of the 276 patients, 21 patients (7.6%) were suspected to have monogenic diabetes; 8 of these patients had clinical MODY, and the remaining 13 patients had other types of monogenic diabetes. Among them, genetic etiologies were identified in 14 patients (5.1%) from 13 families (Table 2, Supplementary Table 1).

**Table 2:** Mutations in genes associated with monogenic diabetes

Gene	Nucleotide change	Amino acid change	Exon/Intron	Segregation data	ACMG/AMP guidelines	Phenotype
*HNF1B*	c.443C > T	p.S148L	1	NA	Pathogenic	MODY 5
*KCNJ11*	c.602G > A	p.R201H	1	NA	Pathogenic	DEND syndrome
*WFS1*	c.2171C > T	p.P724L	8	Sibling	Pathogenic	Wolfram syndrome
*WFS1*	**c.1725_1742del**	**p.G587_G592del**	8	Sibling	Likely pathogenic	Wolfram syndrome
*INSR*	c.3196C > T	p.R1066*	17	Paternal	Pathogenic	Donohue syndrome
*INSR*	c.3614C > T	p.Q1232*	21	Maternal	Pathogenic	Donohue syndrome
*FOXP3*	c.201 + 1G > A	Splice site	1	Maternal	Pathogenic	IPEX syndrome
*SLC2A2*	c.13A > T	p.K5*	1	NA	Pathogenic	Fanconi-Bickel syndrome
*EIF2AK3*	c.1293G > A	p.W431*	6	NA	Likely pathogenic	Wolcott-Rallison syndrome
*CFTR*	c.4056G > C	p.Q1352H	25	NA	Uncertain significance	CFRD
*CFTR*	c.1322 T > C	p.L441P	10	NA	Uncertain significance	CFRD
*MTTL1*	m.3243A > G	Mitochondrial gene	Mitochondrial gene	NA	Pathogenic	MIDD

**Bold,** novel mutation; *ACMG*/*AMP* Interpretation according to the guidelines of the American College of Medical Genetics and Genomics (ACMG) and the Association for Molecular Pathology (AMP) [24], *NA* not available

#### Maturity-onset diabetes of the young

Eight patients were suspected to have clinical MODY (Table 3). Their mean age at diagnosis was 13.5 ± 2.8 years (range, 9.4–18.5 years). The patients were non-obese with a body mass index (BMI) of 19.3 ± 1.3 kg/m^2^ and normal serum C-peptide levels at diagnosis (2.2 ± 1.9 ng/mL). A 14.6-year-old girl was diagnosed with MODY 5 with heterozygous mutations in *HNF1B* (c.443C > T [p.S148L] in exon 2), which was previously reported to be pathogenic [27]. She underwent renal transplantation at the age of 16.1 years owing to chronic renal failure. Rare sequence variants in the genes associated with monogenic diabetes were not identified using whole exome sequencing in the remaining 7 patients.

**Table 3:** Clinical and endocrinological characteristics at diagnosis in 8 patients with clinical MODY

Subject No.	Molecular defect	Sex	Age at diagnosis (year)	Number of generations with DM	BMI	HbA1c,%	Serum C-peptide, ng/mL (0.48–3.3)	Presence of β-cell antibodies (Ab)		Microangiopathy
								GAD Ab, U/mL (0–1)	Insulin Ab, % (0–7)	
1	*HNF1B* c.443C > T (p.S148L)	F	14.6	0	19	6	0.58	NA	NA	Nephropathy
2	ND	F	11.8	3	21.8	8.7	4.4	0.1	4.4	None
3	ND	M	13	3	17.7	14.4	0.37	< 0.1	NA	None
4	ND	M	18.5	0	18.4	7.7	1.4	1.1	6.6	None
5	ND	F	12.3	2	19.2	12.9	1.1	0.94	6.3	None
6	ND	M	12.3	3	20.4	9.5	2.4	0.41	6	None
7	ND	M	15.7	2	18	11.5	1.8	0.28	5.7	None
8	ND	F	9.4	3	19.6	6.7	5.7	1.1	5.1	None

*GAD* Glutamic acid decarboxylase, *ND* Not detected

#### Neonatal diabetes mellitus

Two patients were diagnosed with neonatal DM, one with a transient form caused by paternal uniparental disomy of 6q24 and the other with a permanent form with a heterozygous mutation (c.602G > A [p.R201H]) in *KCNJ11*, leading to developmental delay, epilepsy, and neonatal diabetes (DEND) syndrome [20]. The patient with DEND syndrome was initially misdiagnosed as having type 1 DM; however, insulin therapy was successfully switched to oral sulfonylurea therapy.

#### Wolfram syndrome

Two male siblings were initially misdiagnosed with type 1 DM at the age of 4.9 and 6.1 years, respectively, until urinary incontinence and bilateral optic nerve atrophy occurred. They were compound heterozygous for a known pathogenic c.2171C > T (p.P724L) mutation [28] and a novel c.1725_1742del (p.G587_G592del) mutation in *WFS1*.

#### Donohue syndrome

A male newborn with acanthosis nigricans, hirsutism, high insulin levels, and intrauterine growth retardation was compound heterozygous for c.3196C > T (p.R1066*) and c.3614C > T (p.Q1232*) in the tyrosine kinase domain of the β-subunit in *INSR* [21].

#### Immune dysregulation, polyendocrinopathy, enteropathy, X-linked (IPEX) syndrome

The patient was initially misdiagnosed as having type 1 DM at the age of 11 months. However, he showed unusual clinical features including pure red cell aplasia and membranous glomerulopathy at the age of 39 months, and posterior reversible encephalopathy syndrome after a vaccination against influenza A (H1N1) virus at the age of 11 years. DNA analysis of the *FOXP3* gene identified a splice site mutation of c.201 + 1G > A, which was inherited from his mother [19].

#### Fanconi-Bickel syndrome

A 3-day-old female presented with hyperglycemia, glycosuria, and galactosemia. She failed to grow during the follow-up period, and her liver function deteriorated with micronodular cirrhosis and marked fatty changes on liver biopsy. Homozygous mutation of c.13A > T (p.K5*) in exon 1 was identified by Sanger sequencing of *SLC2A2* [22].

#### Wolcott-Rallison syndrome

A 3-month-old Arab female infant presented with neonatal DM, epiphyseal dysplasia, and liver failure; she was diagnosed with Wolcott-Rallison syndrome caused by the pathogenic homozygous mutation of c.1293G > A (p.W431*) in *EIF2AK3*, which was previously reported to be pathogenic [29].

#### Cystic fibrosis-related diabetes

Cystic fibrosis-related diabetes was detected in two patients with *CFTR* mutations. They showed pancreatitis-related hyperglycemia at the age of 7.7 and 14.2 years, respectively, without β-cell autoantibodies.

#### Maternally inherited diabetes and deafness

Three patients with m.3243A > G mutation in *MTTL1* were diagnosed with maternally inherited diabetes and deafness with a hemoglobin A1c (HbA1c) level of 8.2 ± 2.8% without β-cell autoantibodies. All patients have been treated with insulin injections.

## Discussion

This study described the clinical characteristics of different types of DM based on the experiences of a single tertiary center over the last 20 years. Type 1 DM accounted for most cases (74.6%) of DM in the cohort, followed by type 2 DM (17.8%). Genetic etiologies were confirmed in 14 patients (5.1%) from 13 families. Diverse genetic etiologies are associated with pediatric monogenic diabetes, and extra-pancreatic features were found to be an important clue to the diagnosis of monogenic diabetes.

The frequencies of type 1 DM, type 2 DM, and MODY were 85.6, 10.8, and 1.2%, respectively, in the SEARCH study (USA); on the other hand, these ratios were 95.5, 1.3, and 1.5%, respectively, in the SWEET study (Europe) [30–32]. The diagnosis of type 2 DM (17.8%) and genetically confirmed monogenic diabetes (5.1%) was more common in the present study compared with the previous studies. The variation in the frequencies could be explained by the availability of genetic testing and the prevalence of obesity in the region. Antibody positivity has been reported in up to 15% of patients with type 2 DM, and these autoantibody-positive patients are usually younger, less overweight or obese, and have higher HbA1c levels [33]. However, there were no significant differences in the current study.

Monogenic diabetes comprises various phenotypes including neonatal DM, MODY, and rare syndromic diabetes with extra-pancreatic features including neurological, renal, intestinal, or skeletal abnormalities [6]. Therefore, monogenic diabetes might be initially misdiagnosed as type 1 or type 2 DM prior to the manifestation of extra-pancreatic features, as in the case of the patients with Wolfram syndrome, DEND syndrome, and IPEX syndrome in the present study.

Establishing the etiology of DM is important for therapeutic strategies, the prognosis of chronic complications [14], and appropriate genetic counseling for monogenic diabetes [6]. For example, the molecular diagnosis of monogenic diabetes can lead to changes in treatment, often with improved glycemic control, as some patients with monogenic diabetes carrying mutations in specific genes (e.g., *HNF1A*, *HNF4A*, *KCNJ11*, and *ABCC8*) can be treated with oral sulfonylureas instead of insulin [34].

MODY is defined as an autosomal dominantly inherited familial form of non-autoimmune diabetes due to a primary defect in pancreatic β-cell function with an age of onset before 25 years of age [35]. Mutations in 14 different genes are known to be associated with MODY [https://www.omim.org/entry/606391, accessed on January 2021]. Among them, mutations in *HNF4A*, *HNF1A*, and *GCK* are the most common causes of MODY [36]. The prevalence of MODY has been estimated to be 1–2% of cases of DM [37]. In an Italian study, MODY was the second most prevalent cause (5.5%) of DM after type 1 DM; however, mutations in MODY-related genes were documented in approximately 1.9% of patients [9]. In India, sequence variants in MODY genes were identified in 15–19% of patients with clinically diagnosed MODY in India [38, 39]. Among them, *HNF1A* or *ABCC8* mutations were the most common [39]. The frequency of mutations in the major MODY genes (*HNF4A*, *GCK*, and *HNF1A*) has been shown to be extremely low among Korean patients with MODY [17, 25]. However, with the advent of next-generation sequencing, rate at which MODY is diagnosed using genetic screening has increased. In 28 patients with early-onset diabetes in Korea, four pathogenic or likely pathogenic variants were identified in three patients usingy whole exome sequencing [40]. In targeted panel sequencing, molecular genetic diagnosis was possible in 21.1% of 109 patients who were clinically suspected to have monogenic diabetes [41].

A diagnosis of MODY is dependent on the active referral of patients who are likely to have MODY, which suggests that some patients with MODY remain underdiagnosed [42]. Childhood type 2 DM can be confused with MODY owing to a family history and presenting features, as well as obesity or overweight as a possible confounding factor [11]. Clinically, MODY should be considered in patients with atypical features for type 2 DM, including diabetes onset before the age of 45 years, a normal or low BMI, lack of acanthosis nigricans, and normal serum triglyceride levels and/or normal or elevated high-density lipoprotein cholesterol concentrations [43]. In addition, high-sensitivity C-reactive protein levels are low in *HNF1A*-MODY and can be used to distinguish between *HNF1A*-MODY and type 2 DM [44]. In addition, MODY can be misclassified as type 1 DM, especially in cases involving *HNF1A* mutations [9].

However, MODY can be differentiated from type 1 DM by atypical features of type 1 DM. including the absence of pancreatic autoantibodies, low insulin requirements, evidence of endogenous insulin production with detectable serum C-peptide (> 0.6 ng/mL), and the absence of DKA [43]. An additional, non-invasive 2-h postprandial urinary C-peptide to creatinine ratio (UCPCR) test can be used to distinguish between long-standing type 1 DM and *HNF1A*-MODY and *HNF4A*-MODY. A UCPCR of ≥0.2 nmol/mmol is 97% sensitive and 96% specific for differentiating *HNF1A*- and *HNF4A*-MODY from type 1 DM [45].

A correct diagnosis of MODY is important for the treatment and identification of affected or at-risk family members. Despite the low frequency of MODY among pediatric DM patients, non-obese individuals with a family history of DM and those who lack the clinical characteristics of type 1 DM and type 2 DM should be evaluated for MODY using a high index of suspicion.

This study had several limitations. This study is not a national multicenter study and investigated etiologic distribution of DM in pediatric patients under 18 years of age at diagnosis who were diagnosed at a single tertiary center. Thus, the frequency of specific types of DM may not reflect the actual frequency.

## Conclusions

This study showed that genetically confirmed monogenic diabetes accounted for 5.1% of patients evaluated at a single tertiary center over 20 years. Based on our sample, the frequency of mutations in the major MODY genes appears low among pediatric patients in Korea. Healthcare providers should have a high index of suspicion that diabetic patients with a family history of DM or extra-pancreatic features without β-cell autoantibodies might have monogenic diabetes.

## Supplementary Information

**Additional file 1.**

## Data Availability

The datasets used and/or analyzed during the current study are available from the corresponding author on reasonable request.

## Abbreviations

DEND: developmental delay, epilepsy, and neonatal diabetes
DKA: diabetic ketoacidosis
DM: diabetes mellitus
IPEX: immune dysregulation, polyendocrinopathy, enteropathy, X-linked
MIDD: maternally inherited diabetes and deafness
MODY: maturity-onset diabetes of the young
VUS: variant of uncertain significance
