# Supplementary material for: Etiologic distribution and clinical characteristics of pediatric diabetes in 276 children and adolescents with diabetes at a single academic center
Source: BMC Pediatr. 2021 Mar 4;21:108. doi: 10.1186/s12887-021-02575-6 (PMC7931559; doi:10.1186/s12887-021-02575-6)
Supplement: Supplementary file 1 — Additional file 1. [file 12887_2021_2575_MOESM1_ESM.docx]

| Gene | | *HNF1B* | *KCJ11* | *WFS1* | *WFS1* | *INSR* | *INSR* | *FOXP3* | *SLC2A2* | *EIF2AK3* | *CFTR* | *CFTR* |
| --- | --- | --- | --- | --- | --- | --- | --- | --- | --- | --- | --- | --- |
| Nucleotide change | | c.443C>T | c.602G>A | c.2171C>T | **c.1725_1742del** | c.3196C>T | c.3614C>T | c.201+1G>A | c.13A>T | c.1293G>A | c.4056G>C | c.1322T>C |
| Amino acid change | | p.S148L | p.R201H | p.P724L | **p.G587_G592del** | p.R1066* | p.Q1232* | Splice site | p.K5* | p.W431* | p.Q1352H | p.L441P |
| Variant type | | Missense | Missense | Missense | Deletion | Nonsense | Nonsense | Splice site | Nonsense | Nonsense | Missense | Missense |
| Variant based evidence | |  |  |  |  | NMD |  | Canonical ±1 splice site | NMD | NMD |  |  |
| Functional data (Reference) | | [1] | [2] | [3] | None | [4] | [4] | None | None | None | [5] | [6] |
| Variant impact | | PS1, PP5 | PS3 | PS3, PP5 | PM4 | PVS1,PS3 | PS3 | PVS1, PP5 | PVS1 | PVS1 | PS3, PM1 | PS3 |
| Population database | MAF from GnomAD (overall) | NF | NF | 0.000032 | NF | NF | NF | NF | NF | NF | 0.001 | 0.0000041 |
|  | Score | PM2 | PM2 | PM2 | PM2 | PM2 | PM2 |  | PM2 | PM2 |  |  |
| Computational prediction | Polyphen-2 | Probably damaging | Probably damaging | Probably damaging | ND | ND | ND | ND | ND | ND | Probably damaging | Probably damaging |
|  | MutationTaster | Disease causing | Disease causing | Disease causing | ND | Disease causing | Disease causing | ND | Disease causing | Disease causing | Disease causing | Disease causing |
|  | SIFT | Deleterious | Deleterious | Deleterious | ND | ND | ND | ND | ND | ND | Deleterious | Deleterious |
| Conservation | PhyloP | 9.6 | 7.90 | 7.434 | ND | ND | ND | ND | ND | ND | 7.28 | 2.14 |
|  | PhastCons | 1.0 | 1.0 | 1.0 | ND | ND | ND | ND | ND | ND | 1.0 | 1.0 |
|  | Score | PP3 | PP3 | PP3 |  |  |  |  |  |  | PP3 | PP3 |
| Zygosity | | Heterozygote | Heterozygote | Compound heterozygote (PM3) | Compound heterozygote (PM3) | Compound heterozygote (PM3) | Compound heterozygote (PM3) | Hemizygote | Homozygote (PM3) | Heterozygote | Homozygote (PM3) | Heterozygote |
| Segregation data | | NA | NA | Sibling with same phenotype (PP4) | Sibling with same phenotype (PP4) | Paternal | Maternal | Maternal (PS2) | NA | NA | NA | NA |
| Criteria Summary | | PS1, PM2, PP3, PP5 | PS3, PM2, PP3 | PS3, PM2, PP3-5 | PM2-4, PP4 | PVS1, PS3, PM2-3 | PS3, PM2-3 | PVS1, PS2, PP5 | PVS1, PM2-3 | PVS1, PM2 | PS3, PM1, PM3, PP3 | PS3, PP3 |
| Conclusion | | Pathogenic | Pathogenic | Pathogenic | Likely pathogenic | Pathogenic | Pathogenic | Pathogenic | Pathogenic | Likely pathogenic | VUS | VUS |

**Supplementary Table 1.** Curation of sequence variants identified in patients with monogenic diabetes

Bold, novel mutation; MAF, minor allele frequency; NA, not available; NF, not found; ND, not done; NMD, nonsense-mediated decay; VUS, variant unknown significance

**References**

1. Yorifuji T, Kurokawa K, Mamada M, Imai T, Kawai M, Nishi Y, Shishido S, Hasegawa Y, Nakahata T. **Neonatal diabetes mellitus and neonatal polycystic, dysplastic didneys: Phenotypically discordant recurrence of a mutation in the hepatocyte nuclear factor-1β gene due to germline mosaicism**. *J Clin Endocrinol Metab* 2004, **89**(6):2905-2908.

2. Gloyn AL, Pearson ER, Antcliff JF, Proks P, Bruining GJ, Slingerland AS, Howard N, Srinivasan S, Silva JM, Molnes J, Edghill EL, Frayling TM, Temple IK, Mackay D, Shield JP, Sumnik Z, van Rhijn A, Wales JK, Clark P, Gorman S, Aisenberg J, Ellard S, Njølstad PR, Ashcroft FM, Hattersley AT. **Activating mutations in the gene encoding the ATP-sensitive potassium-channel subunit Kir6.2 and permanent neonatal diabetes**. *N Engl J Med* 2004, **350**(18):1838-1849.

3. Inoue H, Tanizawa Y, Wasson J, Behn P, Kalidas K, Bernal-Mizrachi E, Mueckler M, Marshall H, Donis-Keller H, Crock P, Rogers D, Mikuni M, Kumashiro H, Higashi K, Sobue G, Oka Y, Permutt MA. **A gene encoding a transmembrane protein is mutated in patients with diabetes mellitus and optic atrophy (Wolfram syndrome)**. *Nat Genet* 1998, **20**(2):143-148.

4. Rubio-Cabezas O, Patch AM, Minton JA, Flanagan SE, Edghill EL, Hussain K, Balafrej A, Deeb A, Buchanan CR, Jefferson IG, Mutair A; Neonatal Diabetes International Collaborative Group, Hattersley AT, Ellard S. **Wolcott-Rallison syndrome is the most common genetic cause of permanent neonatal diabetes in consanguineous families**. *J Clin Endocrinol Metab* 2009, **94**(11):4162-4170.

5. Lee J-E, Choi JH, Lee JH, Lee MG. **Gene SNPs and mutations in clinical genetic testing: haplotype-based testing and analysis**. *Mutat Res* 2005, **573**(1-2):195-204.

6. Gee HY, Kim CK, Kim SW,Lee JH, Kim JH, Kim KH, Lee MG. **The L441P mutation of cystic fibrosis transmembrane conductance regulator and its molecular pathogenic mechanisms in a Korean patient with cystic fibrosis**. *J Korean Med Sci* 2010, **25**(1):166-171.
